# Hard to Reach or Just Not Enough? A Narrative Review of Inpatient Tobacco Cessation Programs in Pediatrics

**DOI:** 10.3390/ijerph182413423

**Published:** 2021-12-20

**Authors:** Aysha Jawed, Mandeep Jassal

**Affiliations:** 1 Bloomberg Children’s Center at the Johns Hopkins Hospital, Baltimore, MD 21287, USA; 2 Pediatric Pulmonary, Johns Hopkins University School of Medicine, Baltimore, MD 21287, USA; mjassal1@jhmi.edu

**Keywords:** smoking, tobacco, cessation, quitline, Nicotine Replacement Therapy, pediatric, inpatient

## Abstract

Caregiver smoking is a significant risk factor for children with acute and chronic diseases. Hospitalization presents an opportunity to explore caregiver smoking as a modifiable risk factor during a time of crisis when the motivation to change could be heightened. To date, there has not been a published review on inpatient smoking cessation interventions in pediatrics that focus on supporting caregivers of hospitalized children. The goals of this review were to identify and assess the reach and efficacy of tobacco cessation strategies implemented across inpatient units in pediatrics and mother-baby units. This review also proposes clinical and research implications along with program-building recommendations that can help inform future practice in tobacco cessation. A narrative review of the literature identified 14 peer-reviewed studies that described smoking cessation interventions between 2002 and 2021. There were five randomized controlled trials, seven prospective studies, and one retrospective study. The primary kinds of interventions were counseling to heighten caregiver contemplation to quit (*n* = 12), provision of Nicotine Replacement Therapy (NRT) medications (*n* = 7), and follow-up with the local Quitline (*n* = 12). A diverse range of deliverers implemented interventions across studies. Variation in defining quit attempts along with tobacco reduction and cessation outcomes contributed to mixed findings across studies.

## 1. Introduction

Caregiver tobacco use is a significant risk factor for pediatric acute and chronic diseases as well as a leading cause of environmental tobacco smoke (ETS) exposure among children. Caregivers who smoke or vape tobacco products substantially elevate their child’s risk for adverse health outcomes that include respiratory and ear infections, sudden unexpected infant death, premature death, and asthma exacerbations [1,2,3,4,5,6,7,8,9,10]. Furthermore, caregiver tobacco use also increases the likelihood that their children will initiate smoking using conventional cigarettes or electronic nicotine delivery devices in the future and thereby heighten intergenerational transmission of tobacco use [4,5,8,9,11,12].

The American Academy of Pediatrics supports family-centered care to enhance communication and collaboration among families and healthcare providers to ultimately improve health outcomes for children [13]. Family-centered care also involves viewing the child in the context of the family. Oftentimes, many components of family-centered care are in full effect simultaneously during the time of a child’s hospitalization. Hence, this time offers plenty of opportunities where it may be easier to naturally integrate active discussions on tobacco use with caregivers as part of treatment planning for the child.

Under the concept of guardianship, caregivers bear the primary responsibility of looking out for their children. Optimizing support for caregivers will help them better meet their child’s needs. Time of hospitalization presents a unique point in time to heighten support for caregivers across a range of risk factors including caregiver tobacco use in the context of understanding root causes of chief complaints and presenting symptoms. During this time, caregivers are also outside their natural habitat and this environmental change may present them with a fresh opportunity to observe firsthand and reflect on how their choices, behaviors, and circumstances may have had an adverse impact on their child’s state of health.

There have only been a handful of studies that designed and implemented inpatient tobacco cessation programs for caregivers of pediatric patients and many more for hospitalized adult patients. However, the degree of success across these programs in pediatrics has been mixed likely since there are significantly fewer of them which further makes it hard to create a standardized approach for tobacco cessation in this caregiver population.

To date, an extensive review has not been conducted to consolidate findings among these inpatient programs as the basis to evaluate their effectiveness in achieving ETS reduction or cessation outcomes. The goals of this review are the following: (1) critically examine the methods used to reach and engage caregivers, (2) assess acute and long-term outcomes, (3) reflect on strengths and shortcomings of interventions, and (4) explore potential future research directions.

## 2. Materials and Methods

### 2.1. Search Strategy

A narrative review of peer-reviewed literature on inpatient tobacco cessation interventions among caregivers of hospitalized pediatric patients was conducted in August 2021. The medical, public health, and psychosocial databases reviewed were the following: Medline (Northfield, IL, USA), APA PsychInfo, Cochrane Review, Academic Search Premier, CINAHL, ERIC and EBSCO. Key terms used across searches were the following: variants of smoking and vaping, different kinds of vape products (electronic nicotine delivery systems (ENDS) and e-cigarettes), tobacco, cessation, quitline, quit, stop, hospital, inpatient, neneonate/infant/newborn/baby, child, adolescent, pediatric, caregiver, parent and guardian.

### 2.2. Inclusion Criteria

Peer-reviewed journal articles were included that evaluated any inpatient tobacco reduction and cessation interventions among caregivers of hospitalized pediatric patients. Any studies that did not report on tobacco reduction and cessation outcomes were excluded.

### 2.3. Procedure

Both authors independently reviewed all titles and abstracts across databases. Differences concerning full-text inclusion were resolved through consensus. Both authors then independently abstracted data across all included studies on participant characteristics, intervention characteristics, tobacco cessation outcomes, and other qualitative or quantitative information on the nature and implementation of the intervention. Results were compared and discrepancies were resolved through active discussions.

### 2.4. Ethics

Institutional review board approval was not required for this literature review.

## 3. Results

**Figure 1:**
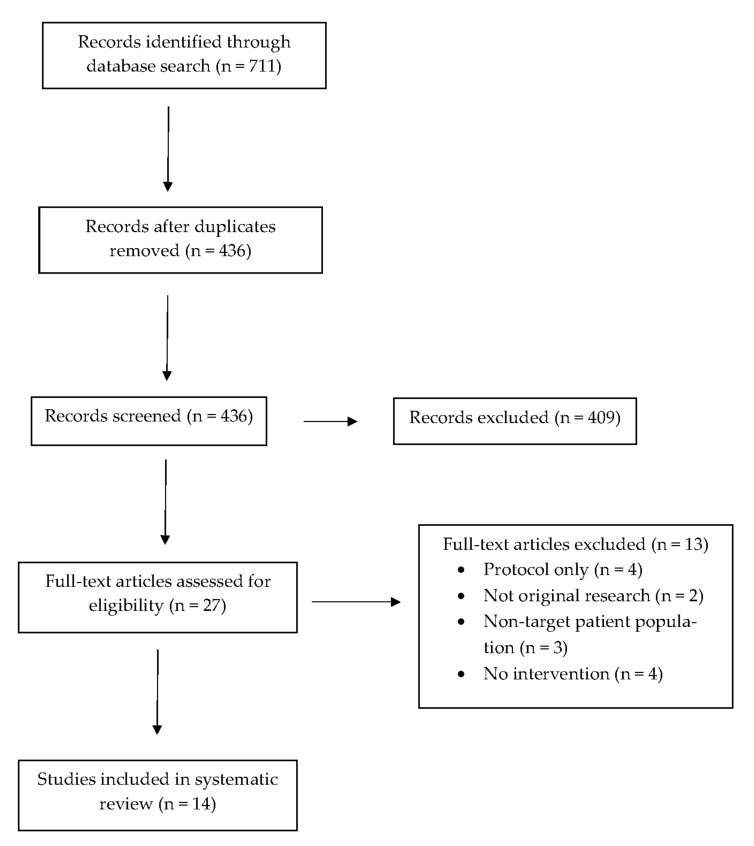
Narrative Review Flowchart.

A cumulative total of 711 records were identified across the databases reviewed from the past 36 years, 275 of these records were duplicates and ultimately excluded. Among the remaining 436 records, 409 of them were subsequently excluded for one or more of the following reasons: (1) were not full-text articles; (2) involved a different target population than caregivers of children; (3) implemented in ambulatory (e.g., primary and subspecialty care clinics, emergency department) or other outpatient and community settings; and (4) did not assess tobacco reduction or cessation outcomes, 27 full-text articles were assessed for inclusion in this narrative review. Among them, 13 were ultimately further excluded for the following reasons: (1) presented only a study protocol; (2) did not involve conducting research to assess for tobacco reduction or cessation outcomes; (3) there was no intervention implemented in the study; (4) the target population comprised a combination of both inpatients and outpatients; and (5) study locations included both inpatient and outpatient settings. Fourteen articles ultimately met the criteria for an inpatient tobacco cessation program for caregivers of pediatric patients as elucidated in Figure 1 [7,14,15,16,17,18,19,20,21,22,23,24,25,26]. IRB approval or an equivalent based on the country was obtained across eleven studies that involved both quality improvement and research [14,15,16,17,19,20,21,23,24,25,26]. A comprehensive breakdown of each study’s participant characteristics, intervention components, and tobacco reduction and cessation outcomes can be found in Table 1.

### 3.1. Sociodemographic Characteristics of Participants

Sociodemographic and illness characteristics assessed at baseline for pediatric patients across most of the studies included race/ethnicity, gender, age, and presenting diagnosis. Sociodemographics obtained for caregivers among nine of the studies included race/ethnicity, gender, age, highest level of education, and health insurance status [7,15,16,19,20,21,22,23,24]. Five studies did not report any information on race/ethnicity of either child or caregiver [17,18,20,21,26]. One study also obtained caregivers’ history of depression and substance use as well as access to primary care [21]. Household income was only assessed in one study [26]. Of note, none of the studies obtained information on household composition, whether caregivers had more than one child and the literacy level of the caregiver.

Caregivers were mainly female across six of the studies [7,21,22,23,24,26]. Children and their caregivers were primarily Caucasian in three studies [7,23,24]. Two of these studies also noted that participants comprised African American, Hispanic, Asian, and other racial/ethnic groups [7,24]. However, in another study, the racial/ethnic composition of children was more diverse and almost evenly split between non-Hispanic black and white followed by Hispanic white and other racial/ethnic groups; 39% of caregivers in this study were non-Hispanic black [22]. In two different studies, children and their caregivers were Chinese and comprised the Han ethnic group [14,16].

Ages of children were not consistently specified across studies but ranged from infancy to 19 years in three of the studies [22,24,26]. Collectively in six studies, the ages of caregivers ranged anywhere from <20 years to ≥40 years [7,20,21,22,23,24].

Many caregivers in six of the studies had obtained at least a high school education [7,16,21,22,24,26]. Health insurance status was also assessed in three studies [20,21,24]. One study that consisted of a sample of 63 children had an almost even split between public (51%) and private (48%) health insurance coverage among the children [24]. In one RCT, 38% of caregivers in the intervention group were uninsured compared to 33% in the control group [21]. In the same study, 43% of caregivers in the intervention group had public health insurance compared to 57% in the control group [21]. In another RCT, 60% of caregivers in the intervention group were insured compared to 80% in the control group [20].

Smoking history was obtained in nine studies and specifically explored caregiver’s frequency of tobacco use, prior quit attempts, number of years smoked, prior use of Nicotine Replacement Therapy (NRT), baseline smoking status, and age when the caregiver began smoking [14,15,16,18,19,20,21,23,24]. Baseline smoking status was mainly determined with the administration of the Fagerstrom Test for Nicotine Dependence in four studies [7,15,20,21]. Another study assessed whether maternal and paternal caregivers had cut back or quit tobacco use at any point during the pregnancy compared to post-birth [15]. Daily tobacco use was challenging to assess in five of the studies that exclusively focused on cigarettes and subsequently excluded possible use of other tobacco products [20,21,22,23,24]. Among three studies, the number of years that caregivers reported smoking also had substantial variation and ranged collectively from 6 years to 24 years [20,21,24]. Unfortunately across studies, the average number of prior quit attempts made by caregivers was hard to quantify given variation in the timeframe constituting a quit attempt. Only one study screened for prior use of NRT and found that 32% of caregivers had previously used NRT products [24]. Lastly, the average score on the Fagerstrom Test for Nicotine Dependence among two studies ranged from 1.2 (low dependence) to 5 (moderate to high dependence), hence indicating that caregivers primarily classified as either low, low to moderate, or moderate to high smokers at baseline [7,21].

### 3.2. Research Teams

Research teams were diverse in composition. One research team consisted of only hospitalists and in fact, conducted the first study that involved having hospitalists take the lead as smoking cessation counselors [21]. Another research team comprised first-year pediatric residents [17]. Two research teams comprised respiratory therapists who were also certified tobacco specialists and cessation coaches [7,26]. A different research team consisted of a social worker and neonatal clinical nurse consultant supervised by the Drug and Alcohol staff within the hospital [18]. Three studies involved a combination of research assistants to obtain sociodemographic information and pediatricians to deliver the cessation intervention [14,16,26]. In one study, research associates conducted every phase of the program implementation [19].

### 3.3. Study Designs

Thirteen studies were quantitative and included prospective cohort, cross-sectional, longitudinal, or randomized controlled trial (RCT) designs [7,14,15,16,18,19,20,21,22,23,24,25,26]. Studies involving RCTs randomized caregivers into intervention and control groups [14,19,20,21,23,26]. One study involved a mixed-methods design [17].

### 3.4. Settings for Recruitment

Hospitals ranged from either freestanding children’s hospitals, children’s centers within academic hospitals, or community hospitals across regions of the U.S., China, and Australia that were geographically diverse [7,14,15,16,17,18,19,20,21,22,23,24,25,26]. Caregivers were recruited from medical/surgical units, newborn nurseries, and neonatal and pediatric intensive care units [7,15,18,19,20,21,22,24,26]. One study recruited maternal caregivers from a postpartum unit [23].

### 3.5. Sample Sizes

Among thirteen studies, sample sizes ranged from 42 to 969 caregivers [7,14,15,16,17,18,19,20,21,22,23,24,26]. In one multi-site collaborative study, 21 hospitals participated and screened 995 medical charts; 45 caregivers in the pre-intervention period and 109 caregivers in the post-intervention period ultimately received tobacco cessation interventions in this study [25]. In another multi-site collaborative study involving 35 hospitals, 2202 charts were reviewed and subsequently 131 caregivers in the pre-intervention period and 205 caregivers in the post-intervention period received tobacco cessation interventions [25].

### 3.6. Inclusion and Exclusion Criteria

Inclusion criteria across four studies specifically included the following: (1) child has one or more respiratory diagnoses, such as asthma, status asthmaticus, bronchiolitis, bronchopulmonary dysplasia, cystic fibrosis, pneumonia, wheezing, reactive airway disease, respiratory distress or failure, stridor, or chronic lung disease; (2) caregiver lives with the child, and (3) caregiver uses one or more tobacco products [7,19,24,26]. Two studies specifically indicated enrolling caregivers of children with neurological, surgical, gastrointestinal, and cardiac conditions [20,21]. The rest of the studies also included caregivers who used tobacco and did not specify parameters surrounding patient diagnosis and living circumstances [14,15,16,17,18,22,23,25].

Exclusion criteria in some of the studies consisted of the following: (1) caregivers who were pregnant and had cardiovascular disease could not receive NRT given that both conditions were contraindications for NRT [21]; and (2) caregivers who were simultaneously enrolled in another smoking cessation program or already receiving pharmacological treatment for nicotine addiction [20].

### 3.7. Theoretical Frameworks

Interventions across studies were grounded in one or more of the following theoretical frameworks: the Health Belief Model [22,23], motivational interviewing [23,24,26], Social Learning Theory [23], Transtheoretical Model (Stages of Change) [21,23,24], either the 3A or 5A model following clinical practice guidelines of Treating Tobacco Use and Dependence [14,16,20,21,23,25,26], or the chronic care model [23].

### 3.8. Screening

Nursing and/or physicians screened for caregiver smoking in the majority of the studies and documented responses in medical charts [7,18,20,21,22,23,24]. Three of the studies solely screened for cigarette use and subsequently did not account for other tobacco products [22,23,24].

All of the studies screened for caregiver smoking through the use of one closed-ended question [7,14,15,16,17,18,19,20,21,22,23,24,25,26]. However, in nine of the studies, the exact wording of the screening question was not clearly noted [7,14,15,16,17,18,19,20,21]. Five studies specified wording for the screening question at the time of admission:

“Does any caregiver who cares for your child smoke cigarettes?” [22]

“Has either parent/guardian smoked a cigarette, even a puff, within the last 12 months?” [23]

“Does either parent smoke cigarettes?” [24]

“Does anyone who lives in your home or who cares for your child smoke?” [26]

In one multi-collaborative study that focused on standardization of screening, the recommended screening question was “Does your child live with anyone who smokes cigarettes or other tobacco products?” [25].

### 3.9. Counseling

Counseling components across studies varied but overall involved assessing the caregiver’s stage of change, motivational interviewing, identification of stressors and triggers, and brainstorming ideas to manage cravings (e.g., through stress balls, exercise, meditation, yoga, journaling, and adult coloring) [7,14,18,19,23,24,25,26]. Seven studies specifically followed the clinical practice guidelines “Treating Tobacco Use and Dependence” as the basis for scheduling the duration of counseling sessions and proposing treatment strategies that considered each caregiver’s stage of change and included discussions on pharmacotherapy [14,16,20,21,23,25,26].

### 3.10. Sign-Out

As part of their interventions, two studies also included a sign-out component given to an existing or assigned primary care provider for the child or caregiver and in some cases to the caregiver’s obstetrician. Sign-out specifically involved faxing documentation about the degree of caregiver’s tobacco use and assessment of his or her readiness to quit, recommendations that caregiver had received from child’s inpatient care providers to help facilitate cessation, the emphasis placed on continued support for the caregiver, and list of NRT products if given or prescribed to caregiver [23,24].

### 3.11. Provision of Resources

#### 3.11.1. NRT Products

In six studies, NRT products were either dispensed or prescribed to caregivers. The quantity of products varied across studies and included a supply of both prescription and over-the-counter gum, patches, and lozenges [7,17,18,19,21,26].

#### 3.11.2. Referral to Quitline

Caregivers in twelve studies were referred to their local quitline or an equivalent telephone counseling service [7,14,15,17,18,19,20,22,23,24,25,26].

#### 3.11.3. Miscellaneous Materials

Educational materials that families received included content on health risks of smoke exposure, ingredients in cigarettes, cost of smoking, and health benefits of quitting [14,16,22,23,24]. One study also involved the development and broadcast of a short video, “Smoking and Kids Don’t Mix,” to explore health beliefs, adverse effects of smoke exposure relevant to children, and recommendations for behavioral and environmental changes that include home and vehicle smoking bans and cessation [22].

### 3.12. Subjective Measures

Six studies involved the dissemination of subjective measures to assess quit attempts, experiences with quitting, and cessation through self-reports provided by caregivers of children [7,15,19,20,21,23]. Across studies, the Fagerstrom Test for Nicotine Dependence was a prevalent subjective measure administered at baseline and one or more follow-up time points to compute a score that classified an individual’s degree of nicotine dependence (low, moderate, high) from a short series of questions on measuring nature and frequency of tobacco use [7,15,20,21].

### 3.13. Objective Measures

In two studies, objective measures involved collecting a baseline saliva sample and 7-day point prevalence of cotinine-verified tobacco abstinence at a follow-up time point. These objective measures were collected in combination with subjective measures to offer a more comprehensive assessment of outcomes [14,23]. Another study also involved assessing cotinine levels in caregivers at the 1-year follow-up [26].

### 3.14. Follow-Up

Follow-up time points post-hospitalization ranged from 1 week to 12 months across studies [7,15,18,20,21,22,23,24,26]. Overall, time points of one week and greater than three months out from hospitalization were poor markers for outcomes. Three studies completed follow-up phone calls after 3 months [20,22,23]. In one study, follow-up discussions on cessation occurred during routine neonatal outpatient clinic visits to account for telephone nonresponders [18]. Findings in one study revealed that there was a substantial loss to follow-up in the long-term (almost 33%) by the 6-month time point [21]. Short-term follow-up revealed marginally greater success in another study as 68% of caregivers were reached during more acute follow-up phone calls at two months after hospitalization [20]. Across three studies, caregivers lost to follow-up were classified as continuing smokers [20,21,22].

### 3.15. Outcome Measures

Quit attempts and cessation were the primary outcomes assessed across studies through self-reports made by caregivers [20,21,22,23,26].

#### 3.15.1. Quit Attempt

Description of quit attempts varied across studies. Five studies defined a quit attempt as any self-reported abstinence that lasted at least one day [14,19,20,23,24].

#### 3.15.2. Quitting

Variations of quitting (quit, cessation) were defined differently in each study as well. Two studies classified quitting as any self-reported abstinence for at least one week [21,24]. In another study, cessation was defined as quitting for at least seven days before completing a follow-up phone call [22].

#### 3.15.3. Secondary Outcome Measures

Other outcome measures included a reduction in the number of cigarettes smoked per day, an increase in the perceived importance of quitting, a report of any contact with the local quitline, and methods used to quit or reduce smoking [20,22].

### 3.16. Data Analyses

All data analyses conducted across studies were quantitative and included normality tests, parametric and nonparametric tests, such as univariate and bivariate analyses, and paired comparison tests to assess outcomes before and after the cessation intervention [7,14,15,16,17,18,19,20,21,22,23,24,25,26]. For six studies with a randomized design, data analyses involved exploring between-group differences across outcomes in the intervention and control groups [14,18,20,22,24,26]. An intention-to-treat analysis was integrated into five studies to account for caregivers lost to follow-up by classifying them as continuing smokers [20,21,22,23,26].

### 3.17. Assessment of Outcomes

Overall across studies, there were mixed findings on quit attempts and cessation. In one study that randomized caregivers into intervention and control groups (21 caregivers in each group), the final quit rate at the time of six-month follow-up was substantially low at 14% in the intervention group [21]. In another study that randomized caregivers into intervention and control groups, 15% of caregivers in the intervention group quit smoking by the time of the 1-year follow-up compared to 8% of caregivers in the control group [26]. A different study that also had a randomized design yielded the greatest caregiver retention at 68% across the entire study duration [20]. In this study consisting of 62 caregivers, 45% reported at least one quit attempt and at two months post-hospitalization, 18% reported quitting [20]. Of note, almost half of the enrolled caregivers perceived smoking cessation as a high priority to preserve the health of their child. In another study involving 167 caregivers, approximately 18% reported cessation at the three-month follow-up [22].

In one study, 35 parents reported making a quit attempt that lasted 24 h and 15 parents reported 7-day abstinence in the 2 months following program enrollment [24]. In a different study, 33% of caregivers reported not smoking at a median time of 6.5 months after initiating transdermal nicotine patch use over a 3 to 9 month follow-up timeframe [18]; 64% of caregivers who quit in the long-term purchased follow-up nicotine patches after their initial supply finished compared to 25% of caregivers who continued to smoke. Additionally, in this same study, the purchase of follow-up patches was a significant predictor of success in quitting. The relapse rate among caregivers was 52%—29 respondents quit initially but 15 of these relapsed. The median time to relapse was 3 weeks with a range of 0.5–12 weeks post-intervention.

In another study, 7% of smokers had quit smoking (defined as having not smoked any cigarettes during the previous 7 days of the follow-up phone call) [16]. A different study involving 35 caregivers had available quit status data after 7 and 8 months which indicated that 39% of caregivers in the newborn nursery quit compared with 0% in the NICU [15]. However, 39% of caregivers in the newborn nursery reduced tobacco use compared with 71% of caregivers in the NICU. One study also involved a group comparison design, most caregivers in both intervention and control groups made at least one quit attempt [19]. Reported smoking declined in the intervention group of caregivers receiving motivational advice and NRT at the first and second follow-up timepoints.

Three studies did not report any reduction or cessation outcomes [7,17,25]. In another study, there was no statistically significant difference in self-reported cessation, cotinine-confirmed cessation, or relapse prevention between the intervention and control groups [23]. Another study also found no significant differences in smoking cessation between the intervention and control hospital caregivers for at least a day (*p* = 0.684) or quitting smoking completely (*p* = 0.510) [14].

There was also variation in the proportion of caregivers who accessed the quitline. One study that enrolled 71 caregivers found that only 7% of caregivers accessed the quitline [24]. The study that had the highest number of caregivers connected with the quitline (greater than 80% in a sample of 101 caregivers) was the first one to implement an inpatient smoking cessation program in a postpartum unit [23]. The rest of the studies had not uncovered how many caregivers had been reached by the quitline [7,14,15,16,17,18,19,20,21,22,25,26].

Among six studies that offered NRT products as part of their cessation intervention, one study reported that more than 25% of caregivers used NRT products post-hospitalization [24]. In another study, 33% of caregivers reported no longer smoking following the use of nicotine patches between 3 to 9 months post-hospitalization (median time of 6.5 months) [18]. Furthermore, 40% of caregivers in this sample reported purchasing nicotine patches after finishing the initial 2-week supply offered at the time of the cessation intervention [18]. Overall, purchase and ongoing use of NRT was a significant predictor of success in quitting across this study as 64% of caregivers who achieved cessation in the long-term purchased additional nicotine patches compared to 25% of caregivers who continued to smoke [18]. As mentioned previously, caregiver engagement with one of the programs implemented was more evident during the two-month follow-up time point than later months [24]. At least one-third of caregivers followed-up with their primary care providers in one study that offered sign-out as part of their inpatient cessation program [24]. However, for caregivers who did not have an assigned primary care provider at the start of this study, hardly any of them followed up with the one assigned to them [24].

Behavior changes among caregivers to reduce secondhand and thirdhand smoke exposure were outcomes achieved alongside quit attempts and cessation outcomes across six studies. These behavior changes included instituting smoking bans in vehicles and homes, handwashing, and changing clothes [14,16,19,22,24,26]. In one study, 60% of caregivers reported smoking inside their home at baseline [24]. However, there was a significant decrease in smoking indoors at the time of 2-month follow-up post hospitalization as only 15% of caregivers reported smoking inside their homes. Furthermore, in this study, 29% of caregivers had rules about no smoking at home at baseline. By the time of the 2-month follow-up, there was a substantial increase in enforcing smokefree rules at home as 71% of caregivers had implemented them. In a different study, smokefree homes increased post-intervention at the time of the 3-month follow-up as 49% of caregivers reported smoking at home compared to 69% of caregivers who did at baseline [16]. Additionally, in this study, there was a nearly 20% increase in smokefree vehicles noted at the 3-month follow-up timepoint as 22% of caregivers reported not smoking inside their vehicles compared to 43% of caregivers who had at baseline. In another study, there was also an increase in initiating smokefree homes post-intervention after 3 months as 55% of caregivers reported not smoking at home compared to 32% of caregivers who had at baseline [14]. Additionally, in this study, smokefree vehicles increased as 76% of caregivers reported that no one had smoked inside their vehicles at the 3-month follow-up timepoint in comparison to 54% of caregivers who had at baseline. Part of this finding could be attributed to the fact that by the time of the 3-month follow-up, 70% of caregivers had enacted smokefree rules in their vehicles compared to 52% of them at baseline.

## 4. Discussion

We conducted a comprehensive narrative review of 14 inpatient tobacco cessation interventions. These studies revealed mixed findings in tobacco reduction and cessation outcomes among caregivers of pediatric patients. Intervention components across most of the studies involved supportive counseling around active contemplation of health behavior change, provision of NRT products, motivational interviewing, and increasing access to local Quitline and additional community resources to promote cessation. However, there were different interpretations across studies about timeframes that constituted successful quit attempts which made it challenging to assess quit attempts as an outcome across different timepoints. Screening questions varied across studies and some of the studies ultimately delimited possible sources of tobacco exposure by only accounting for cigarette smoking. An incomplete understanding exists of the optimal inpatient tobacco cessation intervention, and our review highlights commonalities and differences in interventions that may elucidate the need for novel or personalized programmatic features of inpatient cessation efforts. Thus, we characterized key components of the study design and findings to outline areas for future research to improve this means of addressing pediatric ETS exposures.

### 4.1. Deliverers of Cessation Interventions across Diverse Inpatient Settings

Among all of the studies, there was also diversity in the deliverers of the cessation intervention that involved one or more of the following deliverers: pediatricians, research assistants/associates, nurse practitioners, nurses, respiratory therapists, and social workers [7,14,15,16,17,18,19,20,21,22,23,24,25,26]. This diverse representation further suggests that potentially building a future cessation intervention that accounts for the strengths that each of these deliverers brings to achieving cessation could be promising. It follows that potentially taking a multidisciplinary approach could strengthen the scope and efficacy of implementing a more robust and comprehensive cessation intervention. Diversity of inpatient settings (NICU, medical/surgical units, post-partum units) across all of the studies yields promise in focusing on hospitalization as an optimal time to reach caregivers of pediatric patients as the basis to mediate tobacco use as a risk factor for both acute and chronic illness [7,14,15,16,17,18,19,20,21,22,23,24,25,26].

### 4.2. Electronic-Based Cessation Strategy

One study also emphasized the benefits of utilizing technology through the electronic medical record to provide notification and activate mobilization of cessation resources for caregivers of hospitalized pediatric patients [17]. The EMR system is also a consistent mode of communication among the multidisciplinary care team and could potentially be utilized as a way to identify and facilitate active discussion on follow-up for positive tobacco screens given that it is already embedded into the healthcare system.

### 4.3. Quitline Follow-Up

Only two studies involved follow-up with the quitline directly to assess the frequency of access by caregivers and the exact provision of services offered [15,23]. Only self-reports of quitline access by caregivers measured their degree of engagement with the quitline across the rest of the studies. Having follow-up information from the quitline about the number of contacts, nature of the resources and ongoing counseling offered, text message support, and support groups would provide more descriptive information on caregiver’s access to supportive services and furthermore could offer a more comprehensive assessment of tobacco reduction and cessation outcomes. Finding a way to reduce fragmentation in quitline follow-up could potentially be achieved by exploring possible ways for healthcare institutions to collaborate with the quitline and maintain an open line of communication about referrals made on behalf of caregivers.

### 4.4. Loss to Follow-Up

There was a high loss to follow-up among caregivers across all studies which could potentially be attributed to a myriad of factors including fluctuations in a caregiver’s stage of change suggesting disengagement or ambivalence, competing psychosocial stressors at home, and also the nature of the interventions with respect to their cultural sensitivity, consideration of the psychosocial context, and delivery of content.

Across four studies, caregivers who were lost to follow-up were classified and analyzed as continued smokers which may have contributed to limited statistical significance in the findings and in turn could have underestimated the effects of the cessation intervention [20,22,23,26]. The rest of the studies did not indicate a plan on how to account for caregivers who were lost to follow-up [7,14,15,16,17,18,19,21,24,25]. These findings partly could suggest that the resources in place may not be reaching caregivers successfully given that they originate from different community resources which in turn increases fragmentation in cessation care which is a predictor of loss to follow-up. A future focus could involve creating a comprehensive intervention with resources originating from the same entity. It follows that potentially creating a tobacco reduction or cessation intervention that is embedded in the healthcare system could account for variations in loss to follow-up among caregivers.

### 4.5. Screening for Tobacco Use

Unfortunately, screening for caregiver smoking was also inconsistent among studies. In fact, there is no standardized process across hospitals to document ETS exposure among children who are admitted, which ultimately can result in missing patients and families who could benefit from cessation interventions during hospitalization as a window of opportunity for behavior change. Given that there was no universal way to screen for tobacco use across the majority of the studies, it was challenging to determine the total number of caregivers who smoked or vaped which ultimately underestimated how many caregivers could have been reached by the cessation intervention.

Furthermore, although nursing and medical teams were mainly the first lines of contact to screen across hospitals, the screening question varied and in some cases limited the kind of tobacco use (e.g., only cigarettes) which may have unintendedly resulted in underreporting of actual use. In other cases, some of the screening questions may have had mixed interpretations based on their grammar and syntax which becomes a greater concern among caregivers with low to moderate literacy levels. It is crucial for healthcare institutions to create a standardized screening question that assesses tobacco use and is less likely to result in ambiguity and misinterpretation. Integrating this question into the EMR at the time of admission is one strategy to ensure that it does not get missed across future inpatient cessation interventions.

### 4.6. Discrepancies in Defining Quit Attempts and Cessation

The timeframes specified for a quit attempt and achieved cessation at a minimum also were inconsistent across studies. In fact, it is possible that these timeframes may not have offered realistic space to assess either outcome. These findings are surprising for several studies that had follow-up beyond the 2-month time point since more months out from hospitalization still could not thoroughly account for any increased use or relapse during the time in between follow-ups based on these timeframes. Having a standardized process for accessing quit attempts and cessation will make it easier to measure these outcomes across future studies.

### 4.7. Environmental and Behavioral Changes

As previously noted, there were five studies that involved assessing outcomes pertaining to environmental and behavioral changes from implementing tobacco reduction and cessation interventions. It is crucial to note that smokefree homes and vehicles can be directly linked to quitting. Creating smokefree environments across natural habitats already begins to limit smoke exposure through the enactment and implementation of smokefree rules, changing of clothes, and handwashing. In turn, it follows that it can also further result in health behavior change by reducing the frequency of tobacco use given limited opportunities and spaces for it which could ultimately heighten cessation. This premise can shape the direction of future reduction and cessation endeavors that center on promoting and strengthening smokefree homes and vehicles.

### 4.8. Provision of NRT Products

Disseminating NRT products was not part of the cessation intervention for six studies which could have affected active contemplation among caregivers to quit [14,15,16,20,22,23]. Oftentimes motivation to change is high at times of crisis, and an acute inpatient hospitalization provides an opportunity to take a deeper dive in strengthening caregiver motivation to quit through actively mobilizing resources that could become readily accessible to the caregivers as the basis to support their cessation efforts. Ensuring that NRT products are accessible to caregivers could be a compelling component in future reduction and cessation interventions. Of note, NRT products are likely to reach caregivers faster in healthcare institutions given the increased number of pharmacies that are already within these systems compared to outside community pharmacies.

### 4.9. Attitudes of Clinicians towards the Provision of NRT to Caregivers

Clinicians may have mixed degrees of comfort with providing pharmacotherapy to caregivers, even if it is to mediate health outcomes for their pediatric patients. Their discomfort is likely rooted in the fact that the caregivers are not their immediate patients. However, by adopting a different perspective of the caregiver as a proxy or surrogate for the child, clinicians may develop comfort and confidence in having active discussions with caregivers centered on methods of cessation.

### 4.10. Target Population Considerations

Of note, pediatric oncology patients admitted for cancer treatment were not a focus of any inpatient tobacco cessation programs from this review. Secondhand and thirdhand smoke exposure can be detrimental to these patients and can create more complications with their response to chemotherapy and radiation as well as post-surgical recovery. Furthermore, smoke exposure from tobacco use will also heighten the risks of both cancer reoccurrence as well as the development of new cancers in the future. It is crucial for future programs to carefully consider the unique needs of this specialized patient population and seek to mediate caregiver tobacco use as the basis to optimize positive patient care outcomes from prescribed treatment.

Given that time of hospitalization represents a captive time to reach caregivers of hospitalized children, it may also be a promising window of time to reach hospitalized children across younger and older age groups who are tobacco users. Similar to their caregivers, children may become cognizant during this time of how their tobacco use could have contributed to their presenting problem(s) that necessitated hospitalization. It follows that this time of crisis could be a possible point of intervention for children to heighten their motivation to quit. Furthermore, given that tobacco use is oftentimes initiated during childhood and adolescence, this time could also represent a point of early intervention to cease tobacco use in its nascent stages as the basis to prevent nicotine addiction. Future studies centered on tobacco reduction and cessation could optimize this captive time but also need to design interventions that account for developmental considerations among children. Ensuring that interventions are aligned with the present-oriented mindset of younger populations along with their social identities (e.g., relationships, social networks) could potentially be more relevant and appealing in helping them to quit.

### 4.11. Vaping Cessation Considerations

There were no studies identified that delivered inpatient vaping cessation interventions to caregivers of pediatric patients through this narrative review. In fact, to date, there have been no studies published on vaping cessation for caregivers of children in an inpatient or ambulatory setting. As vaping continues to rise as an increasingly visible public health crisis, it is crucial that the design of future tobacco cessation efforts be inclusive of both conventional and electronic nicotine delivery systems. Part of future vaping cessation endeavors could also involve addressing misinformation about the health consequences from vaping that is oftentimes minimized. Misinformation can originate from a wealth of sources that include tobacco companies and social media influencers that ultimately trickle down to consumers across adult and pediatric populations. Time of hospitalization would also be an optimal and captive time to reach more caregivers directly by implementing a vaping-oriented intervention that could heighten their knowledge and awareness about the harmful effects of vaping as part of increasing their motivation to quit and in turn reduce tobacco exposure for their children as a risk factor for both acute and chronic illness.

As more youth continue to initiate early use of e-cigarettes, electronic nicotine delivery systems (ENDS) could potentially emerge as the category of gateway drugs for precipitating nicotine addiction. Hence, it is crucial for future interventions to account for specific kinds of tobacco use especially involving ENDS given that utilization of these products has increased in prevalence among children as a perceived harm reduction strategy or healthier and safer alternative to conventional tobacco products. It is, therefore, also imperative for future efforts to center on reducing misinformation to children that downplays the risks of vaping which could be just as if not more harmful than conventional tobacco use.

### 4.12. Subjective and Objective Measures

Many of the studies involved eliciting self-reported information from caregivers through subjective measures administered to assess for smoking history, prior quit attempts, degree of nicotine dependence, and tobacco reduction and cessation outcomes which could have resulted in underreporting or over-reporting by the caregivers [7,14,15,16,18,19,20,21,22,23,24,26]. Two studies involved biological or biochemical verification of tobacco use as an objective measure to assess tobacco reduction and cessation outcomes [23,26]. Obtaining a combination of subjective and objective information from caregivers could yield a much more well-informed understanding of the modifiable risk factors that could be targeted for cessation interventions along with critically assessing tobacco reduction and cessation outcomes.

### 4.13. Future Directions of Research

Developing a continuum of care between the inpatient and outpatient spheres would likely support a caregiver’s efforts to sustain motivation to quit. Hospitalization presents a window of opportunity to address caregiver tobacco use as a modifiable risk factor in the context of the child’s overall picture of health. One way to optimize caregiver engagement during this crucial moment in time is to place emphasis around it by engaging the multidisciplinary care team to account for both medical and psychosocial complexities across each intervention component which could further strengthen the efficacy of the intervention. Furthermore, taking a team-based approach can also facilitate securing comprehensive cessation aftercare with wraparound services by tapping into existing resources that will take into account each caregiver’s unique circumstances, goals, values, and priorities. Ensuring that these cessation-oriented resources are utilized to their fullest potential can certainly be challenging given that they originate from different sources. It follows that potentially creating a wraparound program centralized from one source could increase the utilization of cessation resources.

### 4.14. Program-Building Recommendations

The quitline is a community partner across nearly every jurisdiction but unfortunately has had mixed success in reaching adult tobacco users. Inconsistency in the quitline’s efficacy resonates with findings of substantial loss to follow-up months after hospitalization and further emphasizes that there is a missing link between inpatient cessation programs and community resources.

Based on both strengths and limitations inherent in the interventions across studies in this narrative review, creating a more centralized system of care for cessation support that connects both inpatient and outpatient spheres under one umbrella is a proposed solution to mitigating loss to follow-up and improving reduction and cessation outcomes. Furthermore, developing a more sustainable program that is more integrated within the healthcare system could be a possible solution to mediating fragmentation in cessation care. Given demonstrated efficacy in utilizing the electronic medical record in reaching caregivers who screened positive for tobacco use, increased use of healthcare technology presents an opportunity to assess the feasibility of an electronic-based cessation strategy that could involve constructing an algorithm and tobacco cessation order set to screen, treat, and refer caregivers of admitted patients who smoke or vape in a stepwise sequence.

Our future work will involve the development of this integrated institutional approach to promoting collaboration within the hospital as well as with external organizations. However, we recognize that this undertaking will be time-intensive and will necessitate obtaining buy-in from key informants and stakeholders as the first step in streamlining a more harmonized institutional approach in cessation care throughout the healthcare system.

## 5. Limitations of This Narrative Review

This review’s primary limitation is that we did not conduct a systematic review with meta-analyses. The narrative design of this review delimited rigorous examination of study biases and further did not involve conducting composite statistical analyses. In turn, we could not critically assess whether any of the intervention components across studies could be directly related to tobacco reduction or cessation outcomes. In addition, we only reviewed studies published in English which could be another limiting factor of this review.

## 6. Conclusions

From family-centered care, biopsychosocial, and public health perspectives, caregiver tobacco use is a modifiable risk factor to promote positive health outcomes among children, and time of hospitalization presents a golden opportunity to mediate this risk. Unfortunately, there is not a standardized system to screen for caregiver tobacco use across hospitals which unintendedly may underestimate its prevalence. Improving coordination of care between inpatient and outpatient spheres may serve as an invisible bridge to optimize tobacco reduction and cessation success. A wraparound services approach may also mediate the substantial loss to follow-up among caregivers across inpatient tobacco cessation programs in pediatrics.

## Figures and Tables

**Table 1:** Study and Participant Characteristics, Intervention Components, and Primary Outcome Measure.

First Author, Year, Reference. *Location*	Trial Design	Guiding Framework	Participant Characteristics (Sample Size, *% Male*, Age) I: Intervention C: Control	Intervention Components	Dosing	Deliverer of Intervention	Measure of Smoking Cessation	Provider Training: Content and Duration	Findings
Abdullah, 2018 [14] *China*	RCT, quasi-experimental	Transtheoretical Model (Stages of Change), Clinical practice guidelines of Tobacco Use and Dependence, Chronic Care Model	969 *I: 28.6% C: 24.8%* I: 18–24 yrs (11.4%) 25–44 yrs (78.0%) 45 yrs+ (10.6%) C: 18–24 yrs (11.0%) 25–44 yrs (77.4%) 45 yrs+ (11.6%)	Intervention group: counseling, self-help smoking cessation guide, NRT prescription	2 in-person or over-the-phone individual counseling sessions (each between 20–30 min) provided by pediatric resident fellows at initial contact and 1-month follow-up	Pediatric resident fellows at the smoking cessation counseling clinic in the hospital	Chart reviews	Clinical practice guidelines of Tobacco Use and Dependence-2As and R, 5As	Intervention was feasible and acceptable in the delivery of tobacco control assistance. There were no significant differences in smoking cessation between the intervention and control hospital caregivers for at least a day (*p* = 0.684) or quitting smoking completely (*p* = 0.510), increase in initiating smokefree homes post-intervention after 3 months as 55% of caregivers reported not smoking at home compared to 32% of caregivers who had at baseline, smokefree vehicles increased as 76% of caregivers reported that no one had smoked inside their vehicles at the 3-month follow-up timepoint in comparison to 54% of caregivers who had at baseline, 70% of caregivers had enacted smokefree rules in their vehicles at 3-month follow-up compared to 52% of them at baseline
Northrup, 2020 [19] *United States*	RCT, group comparison	Motivational interviewing	32 *I: 6.2% C: 0%* Mean Age: I: 30.6 yrs (SD 9.7) C: 29.9 yrs (SD 4.3)	Intervention group: motivational advice and NRT prescription; Control group: Quitline referral	Intervention group: baseline assessment visit, two in-hospital motivational advice sessions by a research associate, 2-weeks of 14 mg or 21 mg transdermal patches for every smoker in the home, 2 follow-up assessment visits at the hospital or by phone at 2-weeks and 1-month post hospitalization; Control group: baseline assessment visit, smoking fact sheet about the harms of tobacco smoke exposure, Quitline referral, 2 follow-up assessment visits at the hospital or by phone at 2-weeks and 1-month post hospitalization	Research associate	Self-report	Research associate adopted session content from a previous tobacco smoke exposure protocol	Intervention was feasible and acceptable. Most caregivers in both intervention and control groups made at least one quit attempt. Reported smoking declined in the intervention group of caregivers receiving motivational advice and NRT at the first and second follow-up timepoints, self-reported home bans on indoor smoking and car-smoking bans were relatively high at baseline and rose further by the final study visit
Ralston, 2013 [13] *United States*	RCT, group comparison	Clinical practice guidelines of Tobacco Use and Dependence, Transtheoretical Model (Stages of Change)	62 *I: 20% C:34%* Mean Age: I: 29.9 yrs C: 28.3 yrs	Intervention group: counseling, Quitline referral, cessation brochure; Control group: brochure already available to all hospitalized patients and their families	Intervention group: received brief intervention involving counseling < 10 min long, tobacco cessation recommendations from a pediatric hospitalist, contact information for the state Quitline, and a comprehensive smoking cessation brochure created by the American Cancer Society; 2-month follow-up phone call post-hospitalization; Control group: received only an injury prevention brochure that is already given to families of pediatric patients who are hospitalized; 2-month follow-up phone call post-hospitalization	Pediatric hospitalist	Self-report	Assessed caregiver’s degree of nicotine dependence with the Fagerstrom measure in combination with the clinical practice guidelines of Tobacco Use and Dependence as the basis to provide the cessation intervention	18% of caregivers reported quitting at the 2-month follow-up timepoint. 45% of caregivers reported at least one quit attempt at the 2-month follow-up timepoint; 19 caregivers who were lost to follow-up were analyzed as continuing smokers
Ralston, 2008 [21] *United States*	RCT, group comparison	Clinical practice guidelines of Tobacco Use and Dependence, Transtheoretical Model (Stages of Change)	42 *I: 48% C:34%* I: Caregivers- ≥25 yrs (76%) C: Caregivers- ≥25 yrs (71%)	Intervention group: counseling; NRT prescription; Control group: brief counseling and Quitline referral	Intervention group: received extensive smoking cessation counseling from a pediatric hospitalist that involved problem-solving emphasis and lasted > 10 min; caregivers prescribed an 8-week tapering course of nicotine patches beginning with 4 weeks at 21 mg, 2 weeks at 14 mg, and 2 weeks at 7 mg if they smoked > 15 cigarettes/day; for caregivers who smoked < 15 cigarettes/day but still scored at least a 3 on the Fagerstrom, a regimen of 4 weeks of the 14 mg patches followed by 4 weeks of the 7 mg patches were prescribed; 3 and 6-month follow-up phone calls post-hospitalization; Control group: received brief smoking cessation counseling and referral to the state Quitline; 3 and 6-month follow-up phone calls post-hospitalization	Pediatric hospitalist	Self-report	Assessed caregiver’s degree of nicotine dependence with the Fagerstrom measure in combination with the clinical practice guidelines of Tobacco Use and Dependence as the basis to provide the cessation intervention	19% of caregivers in the intervention group and 4.8% of caregivers in the control group were self-reported quitters at the 3-month follow-up timepoint. Final quit rate was 14% in the intervention group at the 6-month follow-up timepoint. 33% of caregivers were lost to follow-up by 6 months and thus analyzed as continuing smokers
Wilston et al., 2021 [26] *United States*	Single-blind RCT	Clinical practice guidelines of Tobacco Use and Dependence-5A model	252 *I: 30.33% (n = 37)* *C: 31.54% (n = 41)* Mean Age: I: 31.8 yrs (SD: 7.3) C: 32.2 yrs (SD: 7.6)	Intervention group: motivational interviewing, education on how to protect children from tobacco exposure, cessation strategies, Quitline referral, NRT provision, follow-up surveys over 1 year; control group: cessation coaches gave brief advice about the importance of quitting smoking and/or reducing their child’s exposure, Quitline referral	Intervention group: cessation coaches offered daily brief (15–30 min) motivational interviewing sessions by phone post-discharge, caregivers received information about protecting children from smoking in the home that included from other smokers or visitors, focused on resolving barriers, identifying triggers, promoting alternatives, and setting a quit date, referral to the state Quitline, 14 days of free dual NRT with patches, lozenges or gum dosed according to number of cigarettes smoked per day, provided standard guidance on NRT use from the package insert, 6-month and 12-month follow-up surveys completed either by phone, online, or in-person; control group: received Ask, Assess, and Advise components of the 5A model, Quitline referral	Diverse cohort of personnel trained to become cessation coaches that included respiratory therapists and research staff	Self-report, cotinine-verified tobacco abstinence	Educational sessions to providers and staff centered on the benefits of reducing tobacco exposure and quitting smoking for the health of their children, 3–4 h online or in-person workshop on motivational interviewing, 1-h tobacco specific motivational interviewing training, ongoing practice sessions addressing different scenarios and assessing skills as well as periodic in-person observation by study leadership	Intervention was feasible and acceptable. 15% quit rate among caregivers in the intervention group and 8% quit rate among caregivers in the control group
Winickoff, 2010 [23] *United States*	RCT, group comparison	Motivational interviewing; Social Learning Theory; Transtheoretical Model (Stages of Change); Health Belief Model; Chronic Care Model; Clinical practice guidelines of Tobacco Use and Dependence-5A model; behavior and systems framework	101 *I: 33% C: 34%* Mean Age: I: 28 yrs C: 30 yrs	Intervention group: motivational interview, counseling, contact information for the Quitline, pamphlet on smoke exposure; Control group: pamphlet with Quitline information	Intervention group: 15-min motivational interview to help caregivers move toward accepting cessation support by enrolling in evidence-based tobacco treatment such as the state Quitline with follow-up feedback from the patient’s pediatrician; 1 individual counseling session; pamphlet about smoke exposure and contact information for the Quitline; 3-month follow-up phone call; Control group: contact information pamphlet for the Quitline; 3-month follow-up phone call	Nurse practitioners and trained research assistants	Follow-up from Quitline, self-report, 7-day point prevalence of cotinine-verified tobacco abstinence at 3 months postpartum	Adapted materials and messages specifically tailored for parental smokers (www.ceasetobacco.org accessed on 22 August 2021); cognitive behavioral and stage-based techniques; Clinical practice guidelines of Tobacco Use and Dependence-5As	Intervention was feasible and acceptable. There was no statistically significant difference in self-reported cessation, cotinine-confirmed cessation, or relapse prevention between the intervention and control groups
Boykan, 2015 [15] *United States*	Prospective, group comparison	Not reported	224 *Newborn Nursery: 25% (n = 46)* *NICU: 23% (n = 11)* Mean Age: Newborn Nursery: 28.6 yrs (SD = 5.7) NICU: 28.6 yrs (SD = 7.5)	Quitline referral	Templates were built within the existing electronic health record to facilitate referral to the New York State Quitline for caregiver smokers of NICU and newborn nursery patients through direct data transfer from the EHR to the Quitline; caregivers were contacted by the Quitline within 3 days after referral and offered a range of quitline services that included telephone coaching and NRT; follow-up with Quitline 7 months post-referral; follow-up phone calls 6–9 months post-referral	Healthcare providers (primarily nurses)	Follow-up from Quitline, self-report	Opt-to-Quit program overview that establishes a systematic policy in which all smokers are offered referral to the New York State Quitline before discharge from a healthcare facility	Intervention was feasible and acceptable. Among the 35 caregivers with available quit status data after 7 and 8 months, there was not a statistically significant difference in quit rates or cutting back. 39% of caregivers in the newborn nursery quit compared with 0% in the NICU. 39% of caregivers in the newborn nursery cut back compared with 71% of caregivers in the NICU. 80% of mothers quit or cut back. 46% of fathers quit or cut back
Huang, 2016 [16] *China*	Prospective, cross-sectional	Clinical practice guidelines of Tobacco Use and Dependence-5A model	107 *62%* Caregivers:18–30 yrs: (42%) 31–44 yrs: (39%) 45+ yrs: (19%)	Counseling and education, pamphlet, poster, sign, and sticker	Focused on the following aspects: (1) health risks of smoking and secondhand smoke exposure; (2) enforcing a strict no-smoking policy at home and in the car; (3) introducing methods and medications for smoking cessation; (4) offering cessation brochures describing the health risks of smoking and children’s secondhand smoke exposure; and (5) providing posters, no-smoking signs, and stickers; 3-month follow-up phone calls	Pediatricians trained as smoking cessation counselors	Self-report	Training consisted of lectures, demonstrations, case reviews, in-class discussions, and role plays. Primary content of the training included epidemiology of smoking and secondhand smoke exposure in China, health hazards of smoking, strategies for smoking cessation including the use of cessation medications and ethical aspects of human research	Intervention was feasible and acceptable. 7% of smokers had quit smoking (defined as had not smoked any cigarettes during the previous 7 days of the follow-up phone call), smokefree homes increased post-intervention at time of 3-month follow-up as 49% of caregivers reported smoking at home compared to 69% of caregivers who did at baseline, there was a nearly 20% increase in smokefree vehicles noted at the 3-month follow-up timepoint as 22% of caregivers reported not smoking inside their vehicles compared to 43% of caregivers who had at baseline
Jenssen, 2016 [17] *nited States*	Single arm prospective and mixed-methods	Clinical practice guidelines of Tobacco Use and Dependence-5A model; health information technology	52 *Not Reported* Not Reported	Counseling, Quitline referral, NRT prescription, behavioral counseling resources	Brief smoking cessation counseling, NRT prescription for either of the following: (1) 2 mg or 4 mg nicotine gum based on whether caregiver smoked first cigarette >30 min after waking up (2 mg) or ≥30 min after waking up (4 mg); or (2) 14 mg or 21 mg nicotine patch based on whether caregiver smoked <10 cigarettes/day (14 mg) or > 10 cigarettes/day (21 mg); Quitline referral placed in discharge instructions; contact information for additional treatment options involving behavioral health resources	First-year pediatric residents	Chart review	Approximately 15–30 min in length and included brief smoking cessation counseling through the 5A model, prescribing NRT including relative contraindications to use and utilization of the parental tobacco clinical decision support tool	Intervention was feasible and acceptable. Limited to process measures of referral and treatment as the outcomes of the study
Ling, 2008 [18] *Australia*	Prospective, longitudinal	Motivational interviewing	42 *Not Reported* Not Reported	Counseling, NRT prescription, smoking cessation information, QUIT program registration	Brief motivational counseling largely provided by a social worker, neonatal clinical nurse consultant who were supported by information, advice, and clinical supervision by the Drug and Alcohol staff within the hospital; 14–21 mg nicotine patches for 2 weeks prescribed by a neonatologist with support from a pharmacist to caregiver based on smoking history; supply of written smoking cessation information (QUIT kits, New South Wales Department of Health, Australia), QUIT program registration (NSW Department of Health, Australia), 3–9 month follow-ups via phone calls or at routine neonatal outpatient clinic visits	Social worker and neonatal clinical nurse consultant	Self-report	Training on behavioral treatments	At a median time of 6.5 months after transdermal nicotine patch use (range 3–9 months), 33% (*n* = 14) caregivers were not smoking. 64% of caregivers who quit long-term purchased follow-up nicotine patches after initial supply finished compared to 25% of caregivers who continued to smoke. Purchase of follow-up patches was a significant predictor of success in quitting. Relapse rate was 52%—29 respondents quit initially but 15 of these relapsed. Median time to relapse was 3 weeks with a range of 0.5–12 weeks after beginning program
Sweeney 2020 [7] *United States*	Prospective, cross-sectional	Cognitive behavioral techniques; coping skills	138 *44% (33)* Age Mean: 31 years	Counseling, NRT prescription, referrals to Quitline and additional community resources	Counseling focused on stressors and triggers, finding alternative ways to manage cravings (stress balls, exercise, meditation, yoga, journaling, adult coloring); provision of NRT that included a combination of over-the-counter NRT (nicotine patches, gum and lozenges) in various doses; referred to outpatient and community programs through the American Lung Association Quitline or state or county department of health; 1-week follow-up phone call post-discharge	Respiratory therapists	Self-report	Training to become certified as tobacco treatment specialists	The intervention was feasible and acceptable; no cessation outcomes reported
Walley, 2015 [22] *United States*	Prospective, cross-sectional	Health Belief Model	167 *30%* 31.5 ± 10.6 yrs	Motivational video, educational materials, Quitline referral	Caregivers viewed a 7-min long motivational video, “Smoking and Kids Don’t Mix” created by an internal hospital team that reviewed adverse health effects of childhood tobacco smoke exposure and recommended behaviors to reduce exposure that included home and vehicle smoking bans and smoking cessation; received written smoking cessation materials; Quitline referral; 1 and 3-month follow-up phone calls to assess knowledge, behavioral changes that included quit attempts, smoking reduction or cessation, and methods used to quit or reduce smoking	Internal hospital team consisting of pediatricians, nurses, and media experts	Self-report	Materials obtained from the Children’s of Alabama Patient Health and Information Center and the American Academy of Pediatrics Julius B. Richmond Center for Excellence	Among the 71 caregivers who were smokers at baseline, 13 of them reported smoking cessation at the 3-month follow-up timepoint. Intervention resulted in behavior changes that ultimately decreased secondhand and thirdhand smoke exposure (e.g., washing hands, changing clothes, initiation of home and vehicle smoking bans)
Walley, 2018 [25] *United States*	Retrospective	Clinical practice guidelines of Tobacco Use and Dependence-5A model and derived 2A and 1 R (ask, advise, and refer) model	BQIP: 21 Hospitals (1869 charts reviewed) SIB: 35 Hospitals (4389 charts reviewed) *Not Reported* Not Reported	Counseling, referrals to community resources	Research teams across both hospital sites received a tobacco change package of interventions that included suggested best practices to increase screening of children for tobacco smoke exposure and provision of tobacco-dependence treatment and referrals for caregivers; counseling; pharmacotherapy; personalized advice to quit smoking; referral to local resources and the Quitline; NRT prescription recommendation	Pediatric hospitalists	Chart review-intervention rate is defined as the rate of documentation of cessation counseling or referral for services in the chart for children with positive tobacco exposure screens	Each hospital site received coaching and feedback. In the BQIP collaborative, the tobacco-dependence treatment interventions recommended were based on the clinical practice guidelines 5A model; a derived 2A and 1 R version was also provided.	Change package interventions were feasible and acceptable. Cessation outcomes were not assessed across both collaboratives.
Winickoff, 2003 [24] *United States*	Prospective, cross-sectional	Transtheoretical Model (Stages of Change); Motivational interviewing	71 *24% (*n* = 17)* 33 ± 9 yrs	Counseling, provision of educational materials, NRT prescription, sign-out to caregiver’s primary care provider, Quitline referral	Counseling that assessed caregiver’s stage of change and involved motivational interviewing; provision of educational materials on smoke exposure, cost of smoking, ingredients in cigarettes, and health benefits of quitting; 1-week supply of NRT products (nicotine gum or patches); 5-day and 10-day follow-up phone calls within 2 weeks of program enrollment; a note faxed to caregiver’s primary care provider about caregiver’s enrollment in the program and sign-out for follow-up by this provider; Quitline referral; 2-month follow-up phone call to assess caregiver’s quit attempts, smoking behaviors, and satisfaction with the program	Counselors	Self-report	The in-hospital counseling session included the techniques of motivational interviewing. Materials provided were from the STOP library that consists of 25 separate 1–2 page sheets of information designed to respond to the specific concerns raised by parents during the interview	Intervention was feasible and acceptable. 35 parents reported having made a quit attempt that lasted 24 h in the 2 months after program enrollment. 15 parents reported 7-day abstinence at 2-month follow-up, 60% of caregivers reported smoking inside their homes at baseline, significant decrease in smoking indoors at time of 2-month follow-up post hospitalization as only 15% of caregivers reported smoking inside their homes, 29% of caregivers had rules about no smoking at home at baseline and by time of 2-month follow-up, there was a substantial increase in enforcing smokefree rules at home as 71% of caregivers had implemented them
